# Cyclic and Macrocyclic Peptides as Chemical Tools To Recognise Protein Surfaces and Probe Protein–Protein Interactions

**DOI:** 10.1002/cmdc.201500450

**Published:** 2015-11-13

**Authors:** Teresa A. F. Cardote, Alessio Ciulli

**Affiliations:** ^1^ Division of Biological Chemistry and Drug DiscoverySchool of Life SciencesUniversity of DundeeJames Black CentreDow StreetDundeeDD1 5EHUK

**Keywords:** chemical probes, chemical tools, cyclic peptides, macrocycles, protein–protein interactions

## Abstract

Targeting protein surfaces and protein–protein interactions (PPIs) with small molecules is a frontier goal of chemical biology and provides attractive therapeutic opportunities in drug discovery. The molecular properties of protein surfaces, including their shallow features and lack of deep binding pockets, pose significant challenges, and as a result have proved difficult to target. Peptides are ideal candidates for this mission due to their ability to closely mimic many structural features of protein interfaces. However, their inherently low intracellular stability and permeability and high in vivo clearance have thus far limited their biological applications. One way to improve these properties is to constrain the secondary structure of linear peptides by cyclisation. Herein we review various classes of cyclic and macrocyclic peptides as chemical probes of protein surfaces and modulators of PPIs. The growing interest in this area and recent advances provide evidence of the potential of developing peptide‐like molecules that specifically target these interactions.

## Introduction

Protein–protein interactions (PPIs) control and regulate cellular processes including enzyme catalysis, cell signalling and development, and protein homeostasis. PPIs strongly influence the abundance and cellular localisation of the proteins involved, and often determine the specificity and fidelity of their function. The importance of PPIs is further underscored by the highly organised and responsive protein networks that regulate most biological processes, which offer many opportunities for therapeutic intervention. In theory, each PPI in such networks may play a role in physiological and pathophysiological states, and could thus represent a prospective drug target with potential therapeutic relevance.^1^ In this context, the development of chemical tools or probes that can help us understand and dissect the mechanisms and biological roles of specific PPIs is of extreme relevance.

In spite of the vast opportunities provided by targeting PPIs, the physicochemical nature of protein–protein interfaces makes it a serious challenge to develop small molecules that bind to these sites and potentially disrupt these contacts. PPIs usually involve flat surfaces of extended area, in contrast to the more traditional binding sites defined at the active sites of receptors or enzymes, which tend to be more buried from solvent and contain well‐defined and deep pockets. Therefore, the reliable identification and development of binding ligands to protein surfaces, whether they be direct or allosteric modulators of PPIs, remain difficult and unsolved problems. However, much progress has been made in recent years in this direction. In particular, it is becoming increasingly clear that the development of drug‐like PPI inhibitors, and small‐molecule ligands to protein surfaces in general, greatly benefits from the availability of a peptidic ligand to that binding site, either from the natural interacting partner or from synthetic sources. Some notable successful examples of using peptides from the natural protein partner as a starting point for drug design include such well‐characterised systems as MDM2–p53^2^ and VHL–HIF1α.^3^ Such peptidic ligands can provide information about the nature and details of the key interactions required to achieve affinity at the targeted binding site, and can furnish crucial displacement tools to ensure the specificity of interactions of the chemical series developed in the drug development process. Peptides also offer an interesting alternative in their own right as PPI modulators, with a number of advantages over nonpeptidic small molecules: biocompatibility, presenting low toxicity to the organism; chemical flexibility, which enhances the capacity to adapt to large and often flexible surfaces; and modularity, which expands structural diversity, thereby facilitating selectivity and high potency.^4^ Additionally, peptides are able to more closely mimic the features of a protein interface, and as such they constitute suitable candidates as chemical tools to target PPIs. On the other hand, the use of peptides as drugs themselves has major drawbacks, especially in comparison with proteins, antibodies, or drug‐like compounds; these drawbacks include low plasma stability, cleavage by cellular proteolytic enzymes, and in vivo clearance by hepatic and renal activities.^5^ Attempts to use short peptides to mimic protein α helices important in a given PPI have had some success, but face limitations mainly due to the sequence and context dependence of peptide helicity, often resulting in peptides that lack the desired helical structure in solution, which is required for productive interaction. This problem has been partially addressed by the peptide‐stapling approach, which was extensively reviewed recently^6^ and is not covered here. In a complementary strategy, nonpeptide compounds have been successfully developed to mimic the key interactions formed by the *i*, *i*+4, and *i*+7 side‐chains of α helices present at PPIs.^7^ However, these approaches have tended to result in poorly soluble compounds that exhibit limited target selectivity and low cellular potency,^8^ and have not been applied across a wide range of biological sequences and PPI targets.

To circumvent these issues and to enable the use of peptides as chemical tools and therapeutic leads, scientists have been creatively modifying biologically active peptides into molecules with more adequate structural features and physicochemical properties.^9^ This review focuses on cyclic and macrocyclic peptide ligands as chemical tools to recognise protein surfaces and for use as chemical probes of PPIs.

## Head‐to‐tail cyclic peptides

**Figure 1:**
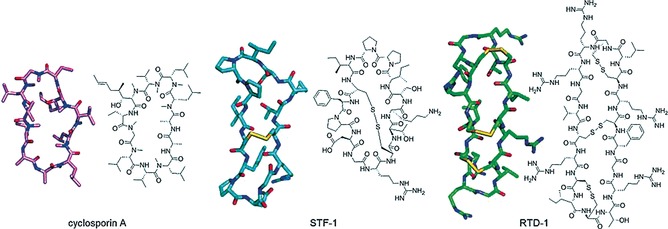
Naturally occurring cyclic peptides: Crystal and chemical structures of cyclosporin A from the fungus *Tolypocladium inflatum* (PDB ID: 2X7K), sunflower trypsin inhibitor (STF‐1) from sunflower seeds (PDB ID: 1JBL), and Rhesus θ defensin 1 (RTD‐1) found in Rhesus macaque (PDB ID: 1HVZ).

Naturally occurring peptides often present cyclic conformations (Figure 1). In the majority of cases, ring structures are formed by disulfide bridges between cysteine residues. However, this is not the only possibility and cyclisation can also be achieved through the formation of amide bonds or aryl–aryl linkages. Examples of such cyclic structures are found from animals to lower plants.^10^ Head‐to‐tail cyclic peptides belong to this latter group, being the most common type, and typically form a ring via amide bond formation between the N‐terminal amine and the C‐terminal carboxylic acid. In discussing head‐to‐tail cyclic peptides, a remark should be made about the Arg‐Gly‐Asp (RGD)‐bearing peptides^11^ as one of the first examples demonstrating the benefits of cyclisation in terms of increasing the stability and affinity of cyclic peptides relative to their linear versions.^12^ Through this section we present and discuss some of the approaches taken to obtain head‐to‐tail cyclic peptides and their applications in modulating PPIs.

The head‐to‐tail cyclisation can be reproduced synthetically by using liquid‐phase peptide synthesis, solid‐phase peptide synthesis (SPPS), or DNA‐programmed chemistry,^13^ and also biosynthetically, with typical examples including phage display approaches^14^ and split‐intein circular ligation of peptides and proteins (SICLOPPS).^15^ Several examples of head‐to‐tail cyclised peptides that effectively target PPIs have been described by the Tavassoli research group. One of the examples is the identification of cyclic peptides that interfere with the HIV Gag protein–TSG101 host protein interaction, an important contact involved in HIV virus outflow.^16^ In this case the authors combined the use of SICLOPPS libraries with a bacterial reverse two‐hybrid system (RTHS)^17^ to identify cyclic peptide disruptors of this PPI. Using this approach against a different target, the same group reported the identification of cyclic hexapeptides that inhibit hypoxia inducible factor (HIF) heterodimerisation with high intracellular activity. Of the four cyclic peptides retrieved from a plasmid‐encoded library of 3.2×10^6^ cyclic peptides, *cyclo*‐CLLFVY (or P1) revealed the capacity to disrupt HIF‐1 heterodimerisation by binding the PAS‐B domain of HIF‐1α, without affecting HIF‐2α.^18^ Using the same approach as in the previous examples, cyclic peptides were used to inhibit the dimerisation of the C‐terminal binding protein (CtBP) transcriptional repressor.^19^ The cyclic peptide CP61 was found to disrupt CtBP homo‐ and heterodimerisation at 20 μm in vitro and to inhibit the cellular functions of CtBPs at 50 μm. Human breast cancer cells treated with the compound showed decreased mitotic fidelity, proliferation, and colony‐forming potential. The authors suggest that a di‐ or tripeptide motif is crucial for the inhibitory activity of the cyclic peptide, with the rest of the peptide acting as a backbone that presents the active motif to its target.^19^

Another recent successful example of head‐to‐tail cyclic peptides as PPI inhibitors was reported by Wu et al., targeting the Ras–effector interaction.^20^ The peptide identified as the most potent binder in vitro (IC_50_=0.83 μm) included unnatural amino acids that were introduced to allow structural diversity and resistance to proteolytic cleavage, but showed poor membrane permeability. This peptide was subsequently optimised into a higher potency Ras inhibitor (IC_50_=0.12 μm) that exhibited high cell permeability and induced apoptosis in cancer cells, making it a useful lead for further development into therapeutic agents.^21^ This work validates the strategy of integrating target‐binding and cell‐penetrating motifs into a single cyclic peptide to develop biologically active inhibitors against other PPIs.

## Side‐chain‐to‐side‐chain cyclic peptides

Side‐chain‐to‐side‐chain cyclisation is another approach that has been extensively explored to obtain cyclic peptides via linkage between amino acid side‐chain groups. In particular, lactam‐bridged peptides have been used to introduce rigidity and defined α‐helical secondary structure in short peptides. Some effective examples of this approach are featured. Using macrolactam constraints between amino acid side‐chains, Mills et al.^22^ reported the first α‐helical peptidomimetics targeting viral RNA. Their synthetic approach involved SPPS with orthogonal Pd^0^‐labile protecting groups on the relevant lactam precursors, followed by on‐resin lactam formation. The tightest binder presented a *K*_D_ value of 40 nm and, interestingly, showed 25‐fold improvement in its binding specificity toward the target, the HIV Rev‐responsive element (RRE) RNA, relative to the corresponding linear precursor. These very encouraging results demonstrate the feasibility of developing peptidic ligands with binding affinities in the nanomolar range. Using a similar approach, i.e., lactam bridges between amino acid side‐chains, the Fairlie research group developed a strategy for inducing peptide helicity by rationally linking together cyclic helical modules as short as five amino acids.^8^ This helix pre‐organisation resulted in significant enhancements in affinity and specificity over unconstrained peptides, and the functional responses were similar to or greater than those of the native proteins from which they were derived. This approach holds potential for rational structure‐based design using native protein structures and combinatorial helix libraries. Furthermore, it successfully demonstrates the ability to downsize proteins from different sources, including bacterial, viral, and human, to short synthetic peptides with strategically enforced water‐stable α‐helical structures.^8^

Computational approaches can aid the rational design of cyclic peptides with biological activities. An example of this is a study targeting the post‐synaptic density protein 95 (PSD‐95), a protein that plays an important role in synaptic plasticity. Molecular modelling of a protein–peptide complex of the third PDZ domain (PDZ3) of PSD‐95 supported the design of inhibitors of this PPI. Based on in silico studies, α‐, β‐, and γ‐amino acids were chosen as bridging elements to tether amino‐ and carboxy‐functionalised residue side‐chains.^23^ The resulting cyclic peptides employed a bis‐carboxylic acid as bridging constraint between two amino‐containing side‐chains from the peptide backbone, and exhibited binding affinities in the single‐ to double‐digit micromolar range.

Different side‐chain‐to‐side‐chain cyclic peptides are those bridged by cysteine disulfide bonds, as illustrated by the following two examples. The first reports cyclic peptides with greater affinity for the CREB binding protein (CBP) bromodomain than their biological ligands, including lysine‐acetylated histones and tumour suppressor p53.^24^ The authors used a target‐structure‐guided and computer‐aided rational design approach to develop a series of disulfide‐linked cyclic peptides for testing in a functional assay. Concerned with the stability of the disulfide bridges inside cells, the authors then modified the most promising peptides, replacing the disulfide by a thioether‐like linker. This work resulted in a series of cyclic peptides in which the best binder exhibited a *K*_D_ value of 8.0 μm, representing a 24‐fold improvement in affinity in comparison with the linear lysine 382‐acetylated p53 peptide.^24^ The second example concerns the development of a cyclic peptide with potential use as a model for the inhibition of the recognition mechanism of HECT‐E3 ligase.^25^ The HECT‐containing E3 ligase Itch mediates the degradation of several substrate proteins, including p63, by recognizing a specific region of p63 that contains a PPxY motif. In this work the authors developed a strategy for the stabilisation of the conformation of an 18‐mer peptide derived from the recognition epitope on p63, and showed that the cyclisation of this peptide enhanced its biological stability and binding affinity. This is a good starting point for the development of drugs that inhibit the Itch E3 ligase complex in vivo.

## Macrocyclics and macrocycle organopeptide hybrids

Merging the biological and synthetic approaches for attaining macrocyclic ligands provides opportunities for ligand diversification in drug discovery. An approach developed by Fasan and co‐workers uses macrocyclic organopeptide hybrids (MOrPHs) incorporating non‐proteogenic synthetic moieties into genetically encoded peptidic frameworks.^26^ MOrPHs are based on the reactivity of intein proteins^27^ and take advantage of the opportunity to introduce bioorthogonal functionalities into proteins by amber stop codon suppression.^28^ These modular assemblies can be easily diversified by altering the nature of the synthetic or biosynthetic precursors. A chemoselective tandem reaction can be performed in the presence of two functional groups with orthogonal reactivity to promote the formation of the organopeptide macrocycle. Using this strategy, several MOrPHs have been prepared that exhibit various ring sizes, structures, and composition and molecular weight, ranging from 700 to 1800 Da.^26^

**Figure 2:**
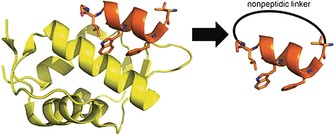
MOrPH molecule design: Crystal structure of HDM2 bound to the peptide PMI (at left) and structural representation of the MOrPH molecule designed based on PMI (right).

Interested in probing the potential of MOrPHs for α‐helix stabilisation and mimicry, the same research group designed MOrPH‐based peptides that target HDM2/X based on a 12‐mer linear peptide isolated by phage display: PMI (Figure 2).^29^ The available crystal structure of PMI bound to HDM2 highlighted the presence of two solvent‐exposed residues as viable side‐chain attachment points for the MOrPH. The resulting molecules are the first examples of side‐chain‐to‐tail peptide cyclisation to aid α‐helix stabilisation and led to the discovery of sub‐micromolar inhibitors of the p53–HDM2/X interaction.^30^ The work also describes the influence of the nonpeptidic moiety in the modulation of the functional, conformational, and stability properties of α‐helical MOrPHs, demonstrating the potential of coupling this approach with a display method as a tool to identify MOrPH compounds with tuned protein binding properties.

In a distinct strategy, using the DNA‐programmed chemistry (DPC) method^31^ for directly translating DNA sequences into small molecules, Seigal et al. developed extensive libraries of DNA‐encoded macrocycles.^32^ The authors describe the construction of macrocycle libraries using 20 different amino acid building blocks for each of the derivatisable positions and five azido‐substituted amino acids in a precise position to allow covalent attachment to the DNA‐encoding template. The screening of these libraries against the BIR2 and BIR3 domains of the X‐linked inhibitor of apoptosis protein (XIAP) led to the identification of novel macrocycles with high affinity. This example shows that producing libraries by DPC is an efficient way to screen for novel macrocyclic inhibitors and to generate meaningful structure–activity relationships (SAR).

Finally, we report thioether macrocyclic *N*‐methyl‐peptide inhibitors of the E6AP E3 ubiquitin ligase identified from a ribosome‐expressed de novo library.^33^ Using translation machinery under reprogrammed genetic code coupled with an in vitro display technique called RaPID (random nonstandard peptides integrated discovery), the macrocyclic *N*‐methyl‐peptides were screened, resulting in a selection of strong anti‐E6AP binders (*K*_D_ values ranging from sub‐nanomolar to single‐digit nanomolar), one of which with the ability to inhibit poly‐ubiquitination of substrate proteins.

## Bicyclic peptides

Bicyclic peptides can be simply defined as cyclic peptides containing two loops. Among possible ways to form such bicyclic structures are side‐chain‐to‐side‐chain linkages on monocyclic peptides as well as covalent linking units attached to three different points on a linear peptide. Bicyclic peptides tend to have a more constrained structure than their monocyclic counterparts. One of the most attractive features of bicyclic peptides is the display of relatively flexible loops constrained to a more rigid central scaffold, which allows closer mimicry of antibodies in terms of their molecular recognition, binding affinity, and specificity properties. In addition, these structural features have been shown to introduce improvements in binding affinities as well as resistance to intracellular degradation and metabolic activities. Several examples have been reported for different and successful constructions of bicyclic peptides, some of which are discussed here.

The introduction of side‐chain‐to‐side‐chain staples in peptide macrocycles can be used as a means of stabilising them. Quartaro and colleagues^34^ reported a strategy for the generation of bicyclic peptides using this approach. The starting point was an 11‐mer disulfide‐bridged macrocycle named G1 that binds the Src‐homology 2 (SH2) domain of growth‐factor‐bound protein 2 (Grb2). G1 had been subjected to several rounds of optimisation, allowing the authors to take advantage of previously known SAR^35^ to help design bicyclic peptide analogues. The approach resulted in a bicyclic macrocycle with a 60‐fold improvement in inhibitory potency and 200‐fold increased selectivity relative to the original peptide after only two rounds of iterative design.^34^ To impart generality and application of this strategy to any given cyclic peptide will, however, require systematic exploration of cross‐link positions to optimise functional properties and to identify ideal staples for each cyclic peptide.

**Figure 3:**
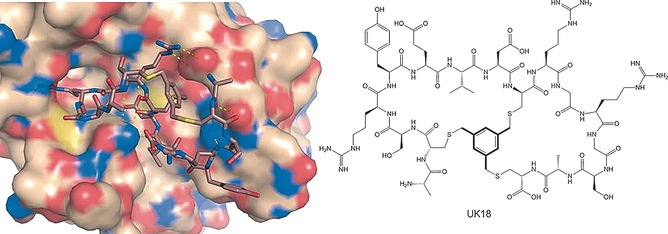
Binding mode and interactions of bicyclic peptides at protein surfaces: A crystal structure of the bicyclic peptide UK18 bound to the urokinase‐type plasminogen activator (uPA) is shown at left; the bicyclic peptide′s conformation reveals extended contacts with the protein surface. At right is the chemical structure of UK18, highlighting the chemical scaffold TBMB.

Substantial research has been directed in the Heinis laboratory toward the development and optimisation of bicyclic peptides to target different proteins. In 2009 Heinis et al.^36^ presented a new strategy for selecting ligands based on bicyclic peptides attached covalently to an organic core scaffold. The peptides contain three reactive cysteine residues, which allow conjugation with 1,3,5‐tris‐(bromomethyl)benzene (TBMB), thereby giving rise to two peptidic loops linked by a mesitylene core. The cysteine residues were spaced apart by a variable number of random amino acids, and the peptides were fused to the phage gene‐3‐protein, permitting the screening of vast libraries of bicyclic peptides with the convenience of phage display. One of the first successful examples described the screening of a library of 4.0×10^9^ different chemically constrained bicyclic peptides to identify a selective and potent (*K*_i_=53 nm) inhibitor (UK18) of human urokinase‐type plasminogen activator (uPA).^37^ The crystal structure of the peptide–protein complex revealed that this peptide (<2 kDa) resembles features typical of PPIs, such as a large interface of interaction with the target and multiple hydrogen bonds and electrostatic interactions from both peptidic loops, contributing to its binding affinity and specificity (Figure 3).^37^ The same group tested the effect of different chemical cores in the variation of the backbone conformation adopted by the peptide loops. Studies with three different scaffolds led to the conclusion that different alkylating reagents impose different constraints on the backbone conformation of bicyclic peptides.^38^ This strong structural effect was not entirely expected due to the relative flexibility of the cysteine side‐chains that react with the chemical core. The authors proposed that this dominant effect is related to the chemical moiety being the central location and forming the branching point of the peptide. Other protein surfaces successfully targeted since then, using the same bicyclic peptide approach, include the human plasma kallikrein (hPK),^39^ the coagulation factor XII,^40^ the epidermal growth factor receptor Her2,^41^ and more recently the Notch1 receptor.^42^ The characterisation of these phage‐selected bicyclic peptides revealed high proteolytic stability relative to linear or monocyclic analogues, and most of them showed good stability in blood plasma.^43^ In addition, to prevent renal clearance, a bicyclic peptide inhibitor of the uPA was successfully conjugated to albumin‐binding peptides, which resulted in an extended half‐life of ∼24 h in mice.^44^

Lian and others developed an approach to obtain bicyclic peptide libraries including non‐proteinogenic (and certain unnatural amino acids) and nonpeptidic building blocks, thereby expanding their structural diversity.^45^ The main difference regarding the previous examples relies on the fact that these libraries are entirely synthetic. The screening of a library containing the trimesic acid as a scaffold against tumour necrosis factor‐α (TNFα) yielded 12 bicyclic peptide hits, of which six showed dissociation constants between 1 and 8 μm. The most active peptide exhibited high selectivity toward the target and a binding affinity of 0.45 μm, representing the most potent non‐protein TNFα inhibitor reported until then.^45^ The same group reported the development of bicyclic peptides with cell permeability by attaching them to cell‐penetrating peptides (CPPs),^46^ which have been widely used to deliver drugs, DNA, RNA, proteins, and nanoparticles into mammalian cells and tissues. Protein tyrosine phosphatase 1B (PTP1B) was selected as a model system, and PTP1B inhibitors were identified in a screen of bifunctional cyclic peptides featuring the CPP motif and a random pentapeptide sequence.^47^ It is suggested that this delivery method combining cell‐penetrating and target‐binding sequences into cell‐permeable inhibitors could be generalisable and extended to other intracellular targets. Taken together, these results encourage future development and optimisation of bicyclic peptides into potential therapeutic drugs.

## Grafted peptides

The use of small proteins that are able to perform the same functions as large proteins has gained popularity in recent years, leading to the expression and even synthesis of modified miniature proteins, called mini‐proteins. Disulfide‐rich mini‐proteins were found to occur naturally in plants (called cyclotides), and due to their size and stability represent an attractive starting point for the development of novel PPI modulators. In fact, grafted peptides are molecules that use cyclotides as scaffolds for the introduction of epitopes with biological activities.^4^ Examples of effective application of grafted peptides for targeting PPIs have been described. Ji et al. engineered a cyclotide that is able to activate the p53 tumour‐suppressor pathway in vivo.^48^ The resulting molecule was found to be a nanomolar‐affinity binder with high stability in human serum. In a different study, the Brunsveld research group developed mini‐proteins as androgen receptor co‐activator mimics through a computational design approach.^49^ The authors subsequently performed SAR studies of mini‐proteins in which synthetic point mutations were made in two of the most potent inhibitors from the previous series, in order to achieve higher potency.^50^ These mutations were designed to maintain the secondary structure of the backbone while increasing the binding affinity through additional favourable interactions at the receptor surface, and resulted in a tenfold gain in potency.

Exciting work published in 2012 by Zoller et al.^51^ reports the combination of phage display and molecular grafting to generate tumour‐targeting mini‐proteins. A double‐disulfide‐stabilised mini‐protein called Min‐23^52^ was used as a molecular scaffold in a phage display combinatorial library, leading to the identification of new disruptors of the interaction between the angiogenesis marker δ‐like ligand 4 (Dll4) and Notch1. To overcome synthetic issues of the peptide hits, the expected binding residues of the Min‐23 phage‐display‐evolved peptide were transferred to the variable loop of STF‐1 (sunflower trypsin inhibitor), a cyclic 14‐residue peptide with a single disulfide bridge. Several mini‐proteins were synthesised and tested for binding, and only one showed specific binding interactions with a *K*_D_ value of 22 nm. This study serves as a proof of concept of applying molecular grafting to the development of new molecular entities for therapeutic use. These mini‐proteins were active and specific in vitro and in vivo and were shown to accumulate in tumour tissue, although further optimisation is warranted.

## Photoswitchable peptide PPI inhibitors

**Figure 4:**
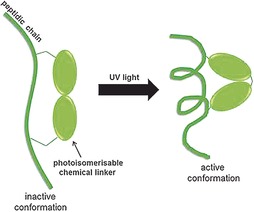
General representation of the photoswitchable peptide approach: The peptide is linked to an isomerisable chemical moiety, which, upon irradiation with light of a certain wavelength, changes conformation and forces the peptide to adopt a specific structure.

The ability to monitor and regulate the activity of PPI inhibitors with spatiotemporal control opens up the possibility to control their kinetics and site of action in cells. Photoswitchable inhibitors are molecules that only adopt their active/inhibitory conformation when exposed to light of a certain wavelength (Figure 4). Therefore, they offer tunable perturbations of biomolecular interactions, allowing regulation of timing and period of activity of the modulators, using light as an external stimulus with high temporal and spatial precision.^53^ This strategy has been applied by several groups, and a few examples are discussed below.

To control clathrin‐mediated endocytosis (CME) in living cells, Nevola et al.^54^ developed photoswitchable peptide inhibitors that target AP2, a complex responsible for internalising cargo in this pathway. Disruptor molecules were designed by starting from a crystal structure of the C‐terminal peptide of the native interaction partner, β‐arrestin, bound to AP2. The binding mode consists of an α‐helical structure with four conserved residues facing the binding pocket. The stability of the secondary structure of peptides is strongly linked with the ability to interact with their target, and this observation led to the hypothesis that one could reversibly regulate the peptides′ affinity by controlling their secondary structure. To achieve this objective, a photoisomerisable cross‐linker, 3,3′‐bis(sulfonate)‐4,4′‐bis(choloroacetamido)azobenzene (BSBBA), was conjugated between pairs of cysteines introduced in the peptide sequence, so that the stability of the helix could be reversibly altered by using 380 nm or 500 nm light. The developed cell‐permeable peptides TL‐1 and TL‐2 were able to disrupt the target PPI in a photocontrolled manner.^54^ Using the same azobenzene linker (BSBBA) acting as a cyclising unit for peptides, by attaching them to the chemical moiety from two different points, a method to screen cyclic peptide libraries by phage display was developed by Heinis and co‐workers.^55^ The authors described the cyclisation of a library of the format ACX_5_CG (where X are random amino acid residues) with BSBBA and screening against streptavidin. The peptides were exposed to UV light prior to affinity panning with the aim of isolating ligands that bind preferentially in the *cis* conformation. The best binder identified showed a *K*_D_ value of 2.2 μm, but the changes in affinity by exposure to UV light did not enhance the affinity drastically. In the future this limitation could be overcome by applying different peptide library formats or different photoswitchable moieties.^55^

The last application described here reports photoactive phosphopeptide mimetics as potent, light‐switchable inhibitors of the protein tyrosine phosphatase PTP1B.^56^ A benzoyl phosphonate containing amino acid, 4‐phosphonocarbonyl phenylalanine, was used to replace the native phosphotyrosine residue. Irradiation of this benzoylphosphonate under the right conditions and subsequent recognition by a phosphotyrosine binding pocket led to photocross‐linking of the target protein. The peptide mimetics synthesised were validated as inhibitors of PTP1B, and it was shown that irradiation with 365 nm light strongly enhanced the inhibitory effects. PTP1B deactivation was found to occur via a radical mechanism and could be reverted by the addition of dithiothreitol (DTT) as reducing agent.

## Summary and Outlook

The relevance of developing peptide‐like molecules that target specific protein–protein interactions has been underpinned by approaches to obtain cyclic peptides and organopeptide hybrids and their respective applications. The achievements in this area, including an increasing number of chemical strategies for constraining peptide secondary structure, will no doubt reinforce the importance of peptide binding epitopes as lead structures.

One of the biggest challenges faced in the field remains surpassing the poor oral bioavailability and liabilities associated with poor pharmacokinetics (PK), pharmacodynamics (PD), and absorption, distribution, metabolism, excretion and toxicity (ADMET) properties that any type of peptidic ligand inherently suffers when used in cellular models and in vivo. Advances have been made toward optimisation of the oral bioavailability and membrane permeability of peptidic ligands in recent years^57^ and corroborate the enhanced properties of constrained peptides over their linear vectors. Improved in vivo lifetime has also been achieved, for example by replacing α‐amino acid residues with homologous β‐residues^58^ or by coupling them to small molecules that bind reversibly to serum proteins.^59^

Successful targeting of PPIs often requires a bilateral relationship between the protein and the ligand involved; therefore, efforts have also been pursued in understanding which characteristics of a protein target might make it more suitable for productive binding by macrocyclic ligands.^60^ This knowledge would definitely provide guidelines for the development of macrocyclic ligands with enhanced structural and physicochemical properties and better bioavailability.

Although there is still a long road ahead, the advances reported herein have increased our understanding of the requirements imposed on peptidic PPI modulators as potential therapeutics. These advancements have significantly boosted the field, as reflected by the increasing number of publications, building confidence that the approach is feasible and likely to deliver major breakthroughs in terms of novel chemical tools and potential new drugs in the near future.
